# # Me Too: Global Progress in Tackling Continued Custodial Violence Against Women: The 10-Year Anniversary of the Bangkok Rules

**DOI:** 10.1177/15248380211036067

**Published:** 2021-08-03

**Authors:** Marie Claire Van Hout, Simon Fleißner, Heino Stöver

**Affiliations:** 1 Public Health Institute, Liverpool John Moores University, United Kingdom; 2 Faculty of Health and Social Work, Frankfurt University of Applied Sciences, Frankfurt am Main, Germany

**Keywords:** gender-based violence against women, GBVAW, prisons, Bangkok Rules, custodial violence

## Abstract

On any given day, almost 11 million people globally are deprived of their liberty. In 2020, the global female population was estimated to be 741,000, an increase of 105,000 since 2010. In order to investigate progress in the adoption of the Bangkok Rules since 2010, we conducted a legal realist assessment based on a global scoping exercise of empirical research and United Nations (UN) reporting, using detailed MESH terms across university and UN databases. We found evidences in 91 documents which directly relate to violations of the Bangkok Rules in 55 countries. By developing a realist account, we document the precarious situation of incarcerated women and continued evidence of systemic failures to protect them from custodial violence and other gender-sensitive human rights breaches worldwide. Despite prison violence constituting a complex and multifaceted phenomenon, very little research (from the United States, Canada, Brazil, Mexico, and Australia) has been conducted on custodial violence against women since 2010. Although standards of detention itself is a focus of UN universal periodic review, special procedures (violence against women) and concluding observations by the UN committees, very few explicitly mentioned women, and the implications of violence against them while incarcerated. We highlight three central aspects that hinder the full implementation of the Bangkok Rules; the past decade of a continued invisible nature of women as prisoners in the system; the continued legitimization, normalization, and trivialization of violence under the pretext of security within their daily lives; and the unawareness and disregard of international (Bangkok and others) rules.

## Background

On any given day, almost 11 million people globally are detained in prisons or other closed settings (Penal Reform International, 2020a). In 2020, the global female population was estimated to be 741,000 and increasing (Penal Reform International, 2020a) with a growth of 105,000 observed in the past decade, particularly evident in Asia (an increase of 50%), Central and South America (an increase of 19%), and Africa (an increase of 24%) (Lenihan, 2020; Penal Reform International, 2020a). Women in custodial settings are a minority and generally imprisoned for less severe, nonviolent crimes, often heavily underpinned by poverty (“crimes of survival”; Penal Reform International, 2020a, 2021a). Their profiles, histories, and pathways into crime and the criminal justice system are distinct from that of men. Many are from racial or ethnic minority backgrounds; they are disproportionately affected by lower socioeconomic status, trauma, histories of interpersonal violence (child, sexual, intimate partner, physical, and emotional), mental illness; and suffer continued exposure to custodial violence from staff or fellow prisoners (Ervin et al., 2020; Jones, 2020; Karlsson & Zielinski, 2020; Lenihan, 2020; Lynch et al., 2012; Penal Reform International, 2017a, 2020a, 2021a; Tripodi & Pettus-Davis, 2013; United Nations [UN] Office on Drugs and Crime, 2008; Wolff et al., 2007). Identified vulnerable groups include those affected by trauma, trafficking and sexual abuse victims, women who use drugs, sexual minorities, young girls, and those with complex comorbid psychiatric and learning disabilities (Bronson et al., 2017; Meyer et al., 2017; Penal Reform International, 2020a; Tripodi & Pettus-Davis, 2013; UN Office of Drug and Crime, 2008). Within the male dominated criminal justice system, women’s gendered and unique health needs are often neglected and ill-resourced, particularly regarding their sexual and reproductive health, mental health, and the treatment of drug dependence (Gadama et al., 2020; Nakitanda et al., 2020; Penal Reform International, 2020a; UN Office on Drugs and Crime, 2008).

The UN Rules for the Treatment of Women Prisoners and Non-Custodial Measures for Women Offenders (*Bangkok Rules*; UN Secretariat, 2010) were adopted by the UN General Assembly on 21 December, 2010. They were developed to support and complement, as appropriate, the 1955 Standard Minimum Rules for the Treatment of Prisoners (UN, 1955), the 1991 UN Basic Principles for the Treatment of Prisoners (UN General Assembly, 1991a), the 1991 UN Standard Minimum Rules for Non-Custodial Measures (*Tokyo Rules;* UN General Assembly, 1991b), and the updated 2016 UN Standard Minimum Rules for the Treatment of Prisoners (*Nelson Mandela Rules*; UN General Assembly, 2016). While the *Mandela Rules* do not specifically refer to women (with Rule 7 referring to self-perceived gender identity), the *Bangkok Rules* as soft law principles lay the foundation for intensified efforts to support women deprived of their liberty (Barbaret et al., 2017; Huber, 2016; Penal Reform International, 2020b). Although essentially underpinned by inherent tensions in human rights for women, “protection versus protectionism” (Berzano, n.d.), they are insufficiently broad regarding gender diversity by adopting a cis-normative stance and excluding transwomen who are at high risk of exposure to sexual violence when detained with males and potential perpetrators of violence against women when placed alongside females (UN Human Rights Office of the High Commissioner, 2016; Van Hout & Crowley, 2021).

Since adoption of the *Bangkok Rules* in 2010, the criminal justice system and its institutions remain largely designed for the dominant male population, and the *Bangkok Rules* are largely implemented in a piecemeal manner, despite observed global increase of women in prison (Lenihan, 2020; Penal Reform International, 2020a). The UN Committee on the Elimination of Discrimination Against Women (CEDAW) has established that discrimination against women encompasses ill treatment that affects women disproportionately, including detention conditions that do not respond to the specific needs of women (referring to the *Bangkok Rules*). The 2015 UN Subcommittee on Prevention of Torture and other Cruel, Inhuman or Degrading Treatment or Punishment (2015) has described concern regarding the situation of women in detention:

the use of sexual violence as torture, including against transgender persons; lack of adequate attention to their right to health care, including sexual and reproductive health rights; the precarious situation of pregnant women and their children living with them; noncompliance with the rule of separation of women and men; shortage of women custody staff; the practice of invasive searches, including in intimate parts of the body, and the use of public nudity; discrimination in access to work, education, and recreational activities; limitations on contact with relatives, including visits by intimates and contact with their children, as a form of punishment.

In addition, although great attention has been focused globally on tackling gender-based violence against women (GBVAW) in the community, and the spotlight has been shone on torture and inhumane treatment in detention itself, very little has been dedicated to gender-specific aspects of countering interpersonal custodial violence against women deprived of their liberty (Penal Reform International, 2017a, 2017b).

The prison system and its authorities have a general obligation to protect prisoners against any type of violence, including excessive use of force (Penal Reform International, 2020c). GBVAW is defined by the UN Declaration on the Elimination of Violence Against Women as:

violence that is directed against a woman because she is a woman or that affects women disproportionately. It includes acts that inflict physical, mental, or sexual harm or suffering, threats of such acts, coercion, and other deprivations of liberty. (Office of the UN High Commissioner for Human Rights [OHCHR], n.d.)

GBVAW represents a human rights breach with states obligations to exercise due diligence to prevent, investigate, and punish these acts, including if perpetrated by officials (see *Article 2 Universal Declaration of Human Rights; Article 1 and 4 c UN Declaration on the Elimination of Violence Against Women*; *Article 7 International Covenant on Civil and Political Rights; Article 1 CEDAW*), most particularly so when experienced as torture or cruel, inhuman or degrading treatment, or punishment within the power-imbalanced custodial setting. Under international law, rape constitutes torture when it is carried out by or at the instigation of or with the consent or acquiescence of public officials, with other forms of sexual abuse violating the prohibition on cruel, inhumane, or degrading treatment or punishment. Other identified forms of custodial GBVAW include strip searches conducted by men or in the presence of men, virginity testing, verbal sexual harassment, use of restraints (including during labor), psychotropic drugs and solitary confinement to control prisoners, inappropriate surveillance by guards during undressing or showers, and the denial of access to medical care by nonmedically trained officials (Amnesty International USA, 2011; McCulloch & George, 2009; Nowak, 2008; Penal Reform International, 2020c; UN Secretary-General, 2006).

The identified threat of ongoing exposure to physical and sexual violence of women by fellow inmates and/or prison staff in custodial settings has continued since 2010 (Penal Reform International, 2021b). Hence, in order to investigate global progress in the adoption of the *Bangkok Rules* since 2010, with view on documenting and assessing the situation of women in prison, the elimination of custodial violence itself and responses to support those women affected, we conducted a legal realist assessment (Leiter, 2015) based on a global scoping review of extant published literature (empirical, humanitarian, and UN Committee reporting). First, we identified all rules of the Bangkok Rules which are directly related to violence. See Table 1.

**Table 1.** Bangkok Rules Relevant to Gender-Based Violence Against Women.

*Rule 6* The health screening of women prisoners shall include comprehensive screening to determine primary health care needs and also shall determine: The presence of sexually transmitted diseases or blood-borne diseases, and depending on risk factors, women prisoners may also be offered testing for HIV, with pre and posttest counselling; Mental health care needs, including post-traumatic stress disorder and risk of suicide and self-harm; The reproductive health history of the woman prisoner, including current or recent pregnancies, childbirth, and any related reproductive health issues; The existence of drug dependency; Sexual abuse and other forms of violence that may have been suffered prior to admission. *Rule 7* If the existence of sexual abuse or other forms of violence before or during detention is diagnosed, the woman prisoner shall be informed of her right to seek recourse from judicial authorities. The woman prisoner should be fully informed of the procedures and steps involved. If the woman prisoner agrees to take legal action, appropriate staff shall be informed and immediately refer the case to the competent authority for investigation. Prison authorities shall help such women to access legal assistance. Whether or not the woman chooses to take legal action, prison authorities shall endeavor to ensure that she has immediate access to specialized psychological support or counseling. Specific measures shall be developed to avoid any form of retaliation against those making such reports or taking legal action. *Rule 8* The right of women prisoners to medical confidentiality, including specifically the right not to share information and not to undergo screening in relation to their reproductive health history, shall be respected at all times. *Rule 10* Gender-specific health care services at least equivalent to those available in the community shall be provided to women prisoners. If a woman prisoner requests that she be examined or treated by a woman physician or nurse, a woman physician or nurse shall be made available, to the extent possible, except for situations requiring urgent medical intervention. If a male medical practitioner undertakes the examination contrary to the wishes of the woman prisoner, a woman staff member shall be present during the examination. *Rule 11* Only medical staff shall be present during medical examinations unless the doctor is of the view that exceptional circumstances exist or the doctor requests a member of the prison staff to be present for security reasons or the woman prisoner specifically requests the presence of a member of staff as indicated in Rule 10, paragraph 2, above. If it is necessary for nonmedical prison staff to be present during medical examinations, such staff should be women and examinations shall be carried out in a manner that safeguards privacy, dignity, and confidentiality. *Rule 12* Individualized, gender-sensitive, trauma-informed, and comprehensive mental health care and rehabilitation programs shall be made available for women prisoners with mental health care needs in prison or in noncustodial settings. *Rule 13* Prison staff shall be made aware of times when women may feel particular distress, so as to be sensitive to their situation and ensure that the women are provided appropriate support. *Rule 19* Effective measures shall be taken to ensure that women prisoners’ dignity and respect are protected during personal searches, which shall only be carried out by women staff who have been properly trained in appropriate searching methods and in accordance with established procedures. *Rule 20* Alternative screening methods, such as scans, shall be developed to replace strip searches and invasive body searches, in order to avoid the harmful psychological and possible physical impact of invasive body searches. *Rule 22* Punishment by close confinement or disciplinary segregation shall not be applied to pregnant women, women with infants, and breastfeeding mothers in prison.	*Rule 23* Disciplinary sanctions for women prisoners shall not include a prohibition of family contact, especially with children. *Rule 24* Instruments of restraint shall never be used on women during labor, during birth, and immediately after birth. *Rule 25* Women prisoners who report abuse shall be provided immediate protection, support, and counselling, and their claims shall be investigated by competent and independent authorities, with full respect for the principle of confidentiality. Protection measures shall take into account specifically the risks of retaliation. Women prisoners who have been subjected to sexual abuse, and especially those who have become pregnant as a result, shall receive appropriate medical advice and counselling and shall be provided with the requisite physical and mental health care, support and legal aid. In order to monitor the conditions of detention and treatment of women prisoners, inspectorates, visiting or monitoring boards or supervisory bodies shall include women members. *Rule 31* Clear policies and regulations on the conduct of prison staff aimed at providing maximum protection for women prisoners from any gender-based physical or verbal violence, abuse and sexual harassment shall be developed and implemented. *Rule 35* Prison staff shall be trained to detect mental health care needs and risk of self-harm and suicide among women prisoners and to offer assistance by providing support and referring such cases to specialists. *Rule 38* Juvenile female prisoners shall have access to age- and gender-specific programs and services, such as counseling for sexual abuse or violence. They shall receive education on women’s health care and have regular access to gynecologists, similar to adult female prisoners. *Rule 41* The gender-sensitive risk assessment and classification of prisoners shall: Take into account the generally lower risk posed by women prisoners to others, as well as the particularly harmful effects that high security measures and increased levels of isolation can have on women prisoners; Enable essential information about women’s backgrounds, such as violence that they may have experienced, history of mental disability, and substance abuse, as well as parental and other caretaking responsibilities, to be taken into account in the allocation and sentence planning process; Ensure that women’s sentence plans include rehabilitative programs and services that match their gender-specific needs; Ensure that those with mental health care needs are housed in accommodation which is not restrictive, and at the lowest possible security level, and receive appropriate treatment, rather than being placed in higher security level facilities solely due to their mental health problems. *Rule 44* In view of women prisoners’ disproportionate experience of domestic violence, they shall be properly consulted as to who, including which family members, is allowed to visit them. *Rule 56* The particular risk of abuse that women face in pretrial detention shall be recognized by relevant authorities, which shall adopt appropriate measures in policies and practice to guarantee such women’s safety at this time. (See also Rule 58 below, with regard to alternatives to pretrial detention.) *Rule 60* Appropriate resources shall be made available to devise suitable alternatives for women offenders in order to combine noncustodial measures with interventions to address the most common problems leading to women’s contact with the criminal justice system. These may include therapeutic courses and counseling for victims of domestic violence and sexual abuse; suitable treatment for those with mental disability; and educational and training programs to improve employment prospects. Such programs shall take account of the need to provide care for children- and women-only services.

We subsequently searched for literature using university databases and scrutinized the OHCHR system for all published domestic reporting to the UN and the UN Committee Against Torture (CAT) and CEDAW observations at the global level since 2010. 101 UN CAT reports and 158 CEDAW reports were scrutinized, with human rights violations pertinent to the identified Bangkok Rules found in 15 UN CAT, 32 UN CEDAW, and 21 other domestic and UN Human Rights Council reports. Third, the academic literature was examined, and we found 23 relevant records where breaches of the Bangkok Rules were evident. In total, 91 documents related directly to violations of the Bangkok Rules in 55 countries. Despite prison violence constituting a complex and multifaceted phenomenon, very little academic research (mostly from the United States, Canada, Brazil, Mexico, and Australia) has been conducted on GBVAW in custodial settings since 2010, with the bulk of the evidence centering on Special Rapporteur and UN (CAT; Human Rights Council, and CEDAW) country-level reporting. Although standards of detention itself is a focus of UN periodic reports, very few explicitly mentioned women, and the implications of violence against them while incarcerated. See Table 2.

**Table 2.** Critical Findings.

Country	Evidence	Number of Documents
North America		
Canada	Strip searches, administrative segregation, overreliance on the use of force and control measures, illegal tranquillizers, denial medical care and support services (Chartrand, 2015), presence of male guards (CEDAW, 2016a)	2
Mexico	Arbitrary detention and illegal detention (Giacomello, 2020)	1
United States	Violence against LGBTI (CAT, 2014a) Violence against women and systemic failure (Bureau of Justice Statistics, 2014; Fuentes, 2014; Kelly et al., 2014; Perez et al., 2014; Seddiqui, 2015; Wolff & Shi, 2011) Violence of constitutional proportions and violation of women prisoners’ rights against cruel and unusual punishment (Harrison, 2020; Kubiak et al., 2017; Stern, 2018)	10
South America		
Argentina	Ill-treatment and invasive body searches (CAT, 2017a; CEDAW, 2016b) Violence, vexatious body searches, solitary confinement, denial of food (Cornell Law School’s Avon Global Center for Women and Justice and International Human Rights Clinic Defensoría General de la Nación Argentina The University of Chicago Law School International Human Rights Clinic, 2013)	4
Bolivia	Non(sex)-segregated prisons, sexual victimization (CAT, 2013a)	1
Brazil	Limited access to justice, sexual violence (CEDAW, 2012a) Multiplicity of hostile and violence acts (Batista et al., 2020; Gama-Araujo et al., 2020; Scherer & Scherer, 2011)	4
Guatemala	High risk of sexual violence facing transgender people (CAT, 2018)	1
Panama	Overcrowding (CEDAW, 2010)	1
Paraguay	GBVAW, especially against transsexual people (CEDAW, 2017a)	1
Uruguay	Conditions in prison, male staff behavior (CEDAW, 2016c)	1
Venezuela	GBVAW (CEDAW, 2014a)	1
Africa		
Benin	Non(sex)-segregated prisons, lack of access to justice (CEDAW, 2013a)	1
Burundi	Overcrowding, poor rations, nonsex separation (CEDAW, 2016d)	1
Equatorial Guinea	GBVAW perpetrated by inmates and guards (CEDAW, 2012b)	1
Eritrea	Sexual violence (CEDAW, 2020)	1
Ethiopia	Horrific conditions, including rapes, ill treatment, torture (CEDAW, 2019a)	1
Gambia	Violence and rape perpetrated by male prisoners and guards (CEDAW, 2015a)	1
Guinea	Nonsex segregation (CAT, 2014b)	1
Mali	Nonsex segregation, GBVAW by police and prison staff (CEDAW, 2016e)	1
Mozambique	Sexual abuse against women and LSBTI people (CEDAW, 2019b)	1
South Africa	Punitive denial of opiate substitution treatment (Hopkins & Marie, 2017; SANPUD, Metzineres & Harm Reduction International, 2019) Consensual sex practices between incarcerated women (Agboola, 2015) Women to women rape (Agboola et al., 2020)	1
Zambia	GBVAW, including rape (CEDAW, 2011)	1
Zimbabwe	Sexual violence and abuse (Zimbabwe & CEDAW, 2020)	1
Europe		
Norway	Risk of sexual violence, lack of health care, lack of drug treatment programs (CEDAW, 2017b)	1
Denmark	Excessive use of solitary confinement, abuse allegations, ill treatment, nonsex segregation (Nowak, 2009) Nonsex segregation and missing protecting measures (Denmark, 2011)	2
Swiss	Lack of guaranteed segregation (Friedrich-Ebert-Stiftung (Bonn), 2015a)	1
France	Overcrowding, inadequate health care access, high risks of suicide, forced psychiatric hospitalization (CEDAW, 2016f)	1
Ireland	inter prisoner violence, including sexual violence, violence perpetrated by staff (CAT, 2017b)	1
UK	GBVAW in police detention (Children’s Rights Alliance for England, 2013)	1
Italy	Lack of health care services, sexual harassment by male guards (CEDAW, 2017c)	1
Montenegro	Lack of health care services, sexual harassment by male guards (CEDAW, 2017d)	1
Bulgaria	Excessive use of force and arrest when in pretrial detention (CAT, 2017c) Inadequate access to health care (Šimonović, 2019)	2
Cyprus	Overcrowding, lack of privacy/health care (CAT, 2019)	1
Turkey	GBVAW in form of sexual violence and torture (CEDAW, 2016g)	1
Ukraine	Use of restraints during medical examination (European Court of Human Rights, 2020)	1
Greek	Risk of violence against refugee, migrant, and asylum-seeking women (CEDAW, 2013b)	1
Spain	Concern for the general situation (UN Human Rights Council. Working Group on Discrimination Against Women in Law and in Practice, 2015) Invasive body searches, excessive prescription of psychotropic drugs (SANPUD, Metzineers & Harm Reduction International, 2019)	2
Asia & Pacific Region		
Australia	Sexual violence, strip searches, insufficient access to health care (CEDAW, 2018)	1
Armenia	Concern about proportionality of sentences for women (UN Human Rights Council. Working Group on Arbitrary Detention, 2010)	1
Cambodia	Violent abuses by prison management, nonsex segregation, male prison guards (CAT, 2011) Poor conditions in pretrial detention (CEDAW, 2019c)	2
China	Overcrowding, risk of violence, concerns regarding extra-legal detention facilities (CEDAW, 2014b)	1
India	Lack of adequate protection measures, lack of medical care (Manjoo & UN Human Rights Council. Special Rapporteur on Violence Against Women, 2014)	1
Indonesia	Sexual abuse in police detention, abuse (CEDAW, 2012c)	1
Japan	Overcrowding, use of restraint (CAT, 2013b)	1
Korea	Vulnerable to sexual violence, no adequate complaint mechanism, death detention, forced abortion, deprived of a fair trial (CEDAW, 2017f)	1
Papua New Guinea	Nonsex segregation in police custody, risk of collective rapes, sexual and other abuses in exchange for favors, forced to perform domestic work, lack of medical care, and basic needs (UN Human Rights Council. Special Rapporteur in Torture and Other Cruel, Inhuman or Degrading Treatment or Punishment, 2011)	1
Tajikistan	Poor conditions (UN Human Rights Committee, 2019)	1
Thailand	Overcrowding, ill-resourced prison settings, invasive body searches (CEDAW, 2017e)	1
Turkmenistan	Violence, physical and psychological pressure, abuse (including rape) (CAT, 2017d)	1
Uzbekistan	Lack of conducive environment lodging complaints, sexual humiliation, sexual violence by public officials, forced sterilization, ill treatment, abuse (CEDAW, 2015b)	1
Middle East		
Afghanistan	Poor conditions, solitary confinement for long periods (CAT, 2017e)	1
Iraq	Allegations of gender-based violence, including torture, ill-treatment, and rape (Friedrich-Ebert-Stiftung (Bonn), 2015b)	1
Israel	Limited access to justice for Palestinian women (CEDAW, 2017g)	1
Libya	Sexual violence from non-state actors and guards (UN Office of the High Commissioner for Human Rights, 2016)	1
Syria	Rape, sexual violence, GBVAW (CEDAW, 2014c)	1
Yemen	Rape, sexual violence, GBVAW (CAT, 2010)	1

*Note*. CAT = UN Committee Against Torture; GBVAW = gender-based violence against women; CEDAW = UN Committee on the Elimination of Discrimination Against Women.

## Adopting the Bangkok Rules and Progress in Tackling GBVAW in Prisons

Empirical studies from the United Kingdom (UK) and the United States reveal that the female prison environment continues to be as emotionally suppressive, conflict-laden, and violent as in male settings, particularly relating to fighting and physical assaults, with inmate-on-inmate violence comparable across male and female facilities, including sexual assaults, transactional sex in return for protection, privilege or basic necessities, and intimate partner violence between prisoners (Ervin et al., 2020; Kottler et al., 2018; Laws, 2019; Thomson et al., 2019). At the global level, women from sexual minorities (included transwomen) continue to be particularly at risk of sexual abuse including rape (Amnesty International USA, 2011; Human Rights Watch, 2018; Van Hout & Crowley, 2021).

In North America, in 2014, the UN CAT reports on violence against LGBTI people which included transwomen in US prisons (CAT, 2014a). Despite the 2003 Prison Rape Elimination Act and the National Standards to Prevent, Detect and Respond to Prison Rape which came into effect in the United States in 2012, academic literature since 2010 highlights systemic failures to protect women and provides continued evidence for official and inmate-perpetrated violence against women (often abusive sexual context but including rape) in prisons (Bureau of Justice Statistics, 2014; Fuentes, 2014; Kelly et al., 2014; Perez et al., 2014; Seddiqui, 2015; Wolff & Shi, 2011). Three US sources report on violence of constitutional proportions and violation of women prisoners’ (including transwomen) rights against cruel and unusual punishment, including a deluge of rape cases, the majority perpetrated by male guards since 2010 (Harrison, 2020; Kubiak et al., 2017; Stern, 2018). In other closed settings in the United States, recent media outputs report on mass hysterectomies carried out on migrants in immigration detention centers and with those women pleading for help on social media being detained in solitary confinement for several days (Andrews & Hackman, 2020; Bryant, 2020; Ghandakly & Fabi, 2021; Lenzer, 2020). Penal Reform International reports on arbitrary detention and illegal detention methods in 2020, including compulsory drug treatment centers where women are detained in Mexico (Giacomello, 2020). Elsewhere, in Canada, one article reports that violent aspects of prison life continue to affect women, in the form of strip searches, administrative segregation, often for long periods, overreliance on the use of force and control measures, restraints with devices such as with “the wrap” or duct tape, and forcible and illegal injection with tranquilizers, denial of medical care and support services (Chartrand, 2015)^.^ The 2016 CEDAW report on Canada criticizes the presence of male guards in female prisons in Canada (CEDAW, 2016a).

In Central and South America, the CEDAW report of Brazil in 2012 reports limited access to justice and sexual violence against women in detention (CEDAW, 2012a). Three empirical studies in Brazil observe the presence of continued power dynamics in female prisons, viewed as sites of exclusion characterized by a multiplicity of hostile and violence acts (Batista et al., 2020; Gama-Araujo et al., 2020; Scherer & Scherer, 2011). The UN CAT reports on femicide and GBVAW in detention in Argentina in 2017 (CAT, 2017a), and the CEDAW reports on ill treatments and invasive body searches of women in detention in 2016 (CEDAW, 2016b). There are reports by the Inter-American Commission on Human Rights and the UN Human Rights Committee of prison policies in Argentina which group the “worst” behaved women together in prisons, with reports of violence, vexatious body searches, solitary confinement, and denial of food (Cornell Law School, Defensoría General de la Nación University of Chicago Law School, 2013; UN Human Rights Committee, 2016). The UN CAT 2013 refers to non(sex)-segregated prisons and the sexual victimization of women in Bolivian detention settings (CAT, 2013a), and the high risk of sexual violence facing trans-people in male prisons in Guatemala in 2018 (CAT, 2018). In Panama, the CEDAW (2010) reports on overcrowding and violence in female prisons. The 2017 report on Paraguay documents GBVAW and especially the sexual abuse of transsexual people in detention settings (CEDAW, 2017a). The CEDAW is concerned about the conditions experienced by women in prison, particularly regarding behavior of male staff in Uruguay in 2016 (CEDAW, 2016c). In Venezuela, the 2014 CEDAW reports on GBVAW in female prisons (CEDAW, 2014a).

In Africa, the African Commission’s Special Rapporteur on Prisons and Conditions of Detention in Africa in 2012 notes no special reference to women’s issues are made and documents the unmet needs of women in the prison setting, risks of exposure to sexual abuse by prison guards, and that the Kampala Declaration ignores the plight of pregnant women (Special Rapporteur on Prisons and Conditions of Detention in Africa, 2012). A 2019 sub Saharan regional assessment highlights the continued vulnerabilities of women prisoners and their experiences of GBVAW, including rape by guards and fellow prisoners (South Africa, Malawi, Zambia, and Nigeria; Van Hout & Mhlanga-Gunda, 2018). Two studies report on invasive searches and the denial of opiate substitution treatment (OST) for incarcerated women who use drugs in South Africa, despite the Special Rapporteur taking note that punitive denial of OST causing withdrawal (known as “arosto” in South Africa) constitutes inhumane and degrading punishment (Hopkins & Marie, 2017; SANPUD, Metzineres & Harm Reduction International, 2019). Studies by Agboola (and colleagues) report on consensual sex practices between incarcerated women (Agboola, 2015) and continued women to women rape in South African prisons (Agboola et al., 2020). The CEDAW 2013 report on Benin documents non(sex)-segregated prisons and the lack of access to justice for female prisoners (CEDAW, 2013a). In Burundi, the 2016 UN CEDAW reported on overcrowding, poor rations, and no sex separation in prisons (CEDAW, 2016d). The CEDAW also reports women being victims of GBVAW by other inmates and guards in Equatorial Guinea in 2012 (CEDAW, 2012b) and exposure of women prisoners to sexual violence in Eritrea in 2020 (CEDAW, 2020). In Ethiopia, the 2019 CEDAW documents on conditions for women in detention settings which include ill treatment, rape, and torture (CEDAW, 2019a). The 2015 CEDAW report on Gambia documents violence and rape perpetrated against women by male prisoners and guards (CEDAW, 2015a). The 2014 UN CAT reports that male and female prisoners are not segregated in prisons in Guinea (CAT, 2014b). The CEDAW also reports on this lack of segregation of the sexes in Mali, and on GBVAW by police and prison staff in Mali in 2016 (CEDAW, 2016e). The 2019 CEDAW report on Mozambique documents sexual abuse against women and LGBTI people in detention (CEDAW, 2019b). In Zambia, the 2011 CEDAW reports on GBVAW, including rape against imprisoned women (CEDAW, 2011), and in Zimbabwe in 2020, the CEDAW documents sexual violence and abuse against women prisoners (Zimbabwe & CEDAW, 2020).

In Europe, the 2017 UN CCEDAW report on Norway takes note of the continued risk of exposure of women in prison to sexual violence and the lack of health care and drug treatment programs for women (CEDAW, 2017b). Building on a report in 2009, by the Special Rapporteur noting excessive use of solitary confinement in Denmark, allegations of women on women abuses, ill treatment of women in custody by males, and the approach not to segregate men and women in prisons (Nowak, 2009), a later investigation concludes in 2011 that given the mixed gender approach in Danish prisons there are continued needs for adequate protection measures (Denmark, 2011). The UN CAT documents the lack of guarantees of segregation in Swiss prisons (Friedrich-Ebert-Stiftung (Bonn), 2015a). In France, the UN CEDAW 2016 reports that female prisons are overcrowded, with inadequate access to health care and with high risk of suicide and forced psychiatric hospitalization (CEDAW, 2016f) The 2017 UN CAT report documents increased interprisoner violence, including sexual violence among female prisoners, and violent assault of staff in Ireland (CAT, 2017b). GBVAW in police detention was also observed in the UK (Children’s Rights Alliance for England, 2013). In Italy (CEDAW, 2017c) and in Montenegro (CEDAW, 2017d) in 2017, CEDAW comments on the lack of access to health services (including OST) and reports of sexual harassment by male guards for women in detention. The 2017 UN CAT reports on the excessive use of force by police against women on arrest and when in pre-trial detention in Bulgaria (CAT, 2017c). Further, in 2019 the Special Rapporteur on Violence against Women reports on inadequate access to gender-specific medical care for women in Bulgarian prisons (Šimonović, 2019). The 2019 UN CAT report on Cyprus describes overcrowding and lack of privacy/health concerns in women’s prisons (CAT, 2019). The UN CEDAW in 2016 documents GBVAW in the form of sexual violence and torture in Turkish prisons (CEDAW, 2016g). In 2016, there was one case against the Ukraine at the European Court of Human Rights regarding the use of restraints of women during medical examination in 2016 (see *Korneykova and Korneykov v. Ukraine*; European Court of Human Rights, 2020). The UN CEDAW reports on conditions and potential risks for exposure to violence experienced by refugee, migrant and asylum seeking women held in Greek reception centers (CEDAW, 2013b). Two 2020 regional European reviews reveal GBVAW in immigration detention settings (Lungu Byrne et al., 2020; Van Hout et al., 2020), with sources from Spanish prisons and Swedish/UK pre-removal settings referring to the denial of medical and mental health care; verbal abuse, random checks by male guards and lack of privacy reported by women (Arshad et al., 2018; Puthoopparambil et al., 2015; Ruiz-Garcia & Castillo-Algarra, 2014; Smith, 2017). The 2015 Report of the Working Group on the issue of discrimination against women in law and in practice on Spain refers to the situation of women in prison (UN Human Rights Council Working Group on Discrimination Against Women in Law and in Practice, 2015). Invasive searches are reported in female prisons in Spain alongside excessive prescription of psychotropic drugs as control measure by authorities (SANPUD, Metzineres & Harm Reduction International, 2019). In Central Asia, the 2010 Working Group on Arbitrary Detention documents its concern on proportionality of sentences for women in Armenia (UN Human Rights Council Working Group on Arbitrary Detention, 2010). Poor conditions are reported in Tajikistan female prisons (UN Human Rights Committee, 2019). In 2017, the UN CAT documents violence, physical and psychological pressures, and abuse (including rape) against women in prison in Turkmenistan (CAT, 2017d). The UN CEDAW reports in 2015 on concerning conditions for women in detention in Uzbekistan and the lack of conducive environment for lodging complaints about their treatment, underpinned by the intersectionality of discrimination, sexual humiliation, threats of sexual violence by public officials when in custody, forced sterilization, ill treatment and abuse of women human rights defenders in detention (CEDAW, 2015b).

Australia reports comparable rates of violence against male and female prisoners (Schneider et al., 2011) but with a continued process to adapt male policies and programs in prisons (Easteal et al., 2015) and a significant reduction in strip searching of women since 2014 (Wachirs et al., 2014). However, the UN CAT reports on sexual violence perpetrated by male prison officers and practices of strip searches, as well as high rates of mental health disorders and insufficient access to care in Australian prisons in 2018 (CEDAW, 2018). In 2017, in Thailand, the CEDAW committee documents the overcrowded and ill-resourced prison settings for women and the practice of invasive body searches conducted on women in prison (CEDAW, 2017e). The UN CAT report on Cambodia, in 2011, reports on violent abuses by prison management committees, the housing of male and female detainees together, and the use of male prison guards to guard female detainees due to limited staff (CAT, 2011) and in 2019 documents very poor congested conditions for women, including the detention of women in pre-trial detention mixed with convicted offenders (CEDAW, 2019c). The 2012 CEDAW report on Indonesia notes a concerning lack of protection to women in custody, reports of sexual abuse of women in police detention, and challenges in the disclosure by women of such abuses (CEDAW, 2012c). The Special Rapporteur on torture reports substandard conditions and abuses against detained women in Papua New Guinea in 2011. The report describes how women are often not separated from men in police custody, not protected from male inmates (at risk of collective rapes); are in danger of sexual and other abuses in exchange for favors or release from police custody, forced to perform domestic work for officers, including the collecting of male detainees bags and bottles filled with urine and excrement; and with severe lack of access to medical care and basic needs (UN Human Rights Council Special Rapporteur on Torture and Other Cruel, Inhuman or Degrading Treatment or Punishment, 2011). In South Asia, the 2014 Special Rapporteur on Violence Against Women notes a significant lack of adequate protection measures to ensure safety of female inmates, including from gender-related killings, and lack of access to essential medical care in India (Manjoo & UN Human Rights Council Special Rapporteur on Violence Against Women, 2014). In East Asia, the 2014 CEDAW report on China documents the increase of women in detention, overcrowding contributing to risk of violence, and presence of extra-legal detention facilities (“black jails”; CEDAW, 2014b). In Korea, the 2017 CEDAW report documents the grave situation of women in detention, who are particularly vulnerable to sexual violence, including rape by State officials; the absence of adequate, independent, and confidential complaint mechanisms; the detention of repatriated women on the criminal charge of “illegal border crossing” and who are “in addition to suffering sexual violence, are at risk of death in detention, subjected to forced abortions and deprived of their right to a fair trial” (CEDAW, 2017f) . In 2013, the UN CAT reports on overcrowding in Japanese women’s prisons and the use of restraints (*Type II* handcuffs and strait jackets) (CAT, 2013b).

With regard to the Middle East, the UN CAT documents allegations of gender-based violence, including torture, ill treatment and rape, against women in detention in Iraq in 2015 (Friedrich-Ebert-Stiftung (Bonn), 2015b). There are reports about rape, sexual abuse, and GBVAW in female prisons in Yemen in 2010 (CAT, 2010) and in Syria from non-state-armed groups as well as from forces of the government in 2014 (CEDAW, 2014c). In Libya, the OHCHR reports about sexual violence against women in detention from guards as well as from non-state actors in 2016 (UN OHCHR, 2016). In 2017, the CEDAW reports on the limited access to justice for Palestinian women in detention in Israel (CEDAW, 2017g). The UN CAT documents poor conditions in female prisons and the use of solitary confinement for long periods in prisons in Afghanistan in 2017 (CAT, 2017e).

## Conclusive Remarks

Although it is beyond the scope of this global legal realist assessment to engage in a very detailed country-level review, we wish to highlight the continued breaches of the *Bangkok Rules* at the global level as they pertain to the conditions of women in detention since adoption, particularly the prevention of and protection from custodial violence when deprived of their liberty. Gender inequity and inequality is pervasive. Although custodial violence in essence violates the internationally recognized prohibition on cruel, inhuman or degrading treatment or punishment, it remains a largely hidden and sensitive topic for both genders when deprived of liberty, with insufficient surveillance of the issue, coercion threatening disclosure (particularly for women), very low rates of perpetrator accountability, and scant prevalence data available at the global level (Amnesty International USA, 2011).

We document the precarious situation of women in prisons, and continued evidence of systemic failures to protect them from custodial GBVAW and other gender-sensitive human rights breaches worldwide, and take note of the dearth of information in many countries worldwide. We highlight three central aspects that hinder the full implementation of the Bangkok Rules: the past decade of continued invisible nature of women as prisoners in the system; the continued legitimization, normalization, trivialization of violence under the pretext of security within their daily lives; the unawareness and disregard of international (Bangkok) rules; and the task to organize different modes of incarceration environment for (female) prisoners who committed nonviolent crimes. Human rights violations encountered by women in the criminal justice and penal systems continue worldwide. Many countries have not fully adopted the Bangkok and Tokyo Rules, leading to congestion and overcrowding in female prisons, lack of protection against violence, particularly when housed in nonsegregated prisons, either perpetrated by officials or by fellow inmates (of both genders), use of psychotropic and physical restraints, arbitrary detention and solitary confinement, and the lack of full access to gender specific medical care, trauma-informed and trauma-responsive mental health supports, and drug treatment (for instance, OST). Inadvertently, our realist account highlights the continued lack of resourcing of female prisons, lack of implementation of noncustodial sentencing for minor and nonviolent offenses, lack of consideration of GBVAW, exploitation and trauma-related pathways into crime (largely poverty or drug-related), and overall lack of oversight in disclosure and penal complaint mechanisms where GBVAW is perpetrated in the closed setting.

These insights give a well-founded basis for relevant UN agencies (UN Women, UN Office of Drug and Crime, UN Development Program, UNAIDS, and others) and the World Health Organization to provide technical assistance and promote further improvements and penal reforms worldwide. Moreover, this gives a substantiated starting point for human rights organizations such as Amnesty International, the Howard League for Penal Reform, Penal Reform International, and Harm Reduction International to appoint targeted and fitting actions to reduce GBVAW in custodial settings. See Table 3.

**Table 3.** Global Implications for Penal Reform and Monitoring of Standards.

Penal policy
Address the invisible nature of women in prison and correctional policies at the government and regional levels. Enhance visibility of the Bangkok Rules and the rights assurances of women in policy and regional reports. Strive to ensure sex segregation, minimum standards of care and reasonable safe accommodation are provided. Strive to eliminate all forms of custodial violence.
Technical assistance for enhanced prison systems
Address the invisible nature of women in prison and correctional procedures through staff training and awareness raising. Support vigilance against all forms of custodial violence in practice and facilitate disclosure for those affected. Ensure all who work in the custodial setting are aware of women’s exposure to GBVAW, exploitation, and trauma-related pathways into crime (largely poverty or drug-related) Ensure that incarcerated women have access to gender-specific medical care, trauma- informed, and trauma-responsive mental health supports. Ensure that noncustodial sentences are applied where possible for minor or nonviolent offenses, alongside other prison decongestion measures.
Research, surveillance, and monitoring
Encourage continued research activity in the field of prison health worldwide. Encourage continued independent inspections, monitoring, and surveillance of prison standards worldwide.

Further, we wish to underscore how this neglect not only constitutes grave human rights abuses but also fuels self-harm, suicide, psychiatric disorders and deaths, and the spread of disease (HIV, Hepatitis C) bridging between prisons and communities. Addressing disease hinges on prison system approached and parameters to address physical and sexual violence in prisons, trauma-related mental health issues, and unsafe injecting of drugs. UN reporting continues to highlight such issues globally where women are discriminated and treated in an unequal manner, alongside the dearth of academic research and access of research teams into prisons (Mhlanga-Gunda et al., 2019). It is further lamentable that despite global prison release schemes during COVID-19 that women including those convicted on minor, nonviolent or drug offenses have been largely overlooked, thereby exposing them to continued violence, trauma, and harm (Penal Reform International, 2020d; Van Hout, 2020).
